# Calanoid copepod zooplankton density is positively associated with water residence time across the continental United States

**DOI:** 10.1371/journal.pone.0209567

**Published:** 2019-01-09

**Authors:** Jonathan P. Doubek, Cayelan C. Carey, Michael Lavender, Amanda K. Winegardner, Marieke Beaulieu, Patrick T. Kelly, Amina I. Pollard, Dietmar Straile, Jason D. Stockwell

**Affiliations:** 1 Virginia Tech, Department of Biological Sciences, Blacksburg, Virginia, United States of America; 2 Queen’s University, Biology Department, Biosciences Complex, Kingston, Ontario, Canada; 3 McGill University, Department of Biology, Montreal, Québec, Canada; 4 Université de Sherbrooke, Département de Génie Civil, Sherbrooke, Québec, Canada; 5 Biology Department, Miami University, Oxford, Ohio, United States of America; 6 Office of Water, U.S. Environmental Protection Agency, Washington, D.C., United States of America; 7 Limnological Institute, University of Konstanz, Konstanz, Germany; 8 University of Vermont, Rubenstein Ecosystem Science Laboratory, Burlington, Vermont, United States of America; University of Connecticut, UNITED STATES

## Abstract

Inherent differences between naturally-formed lakes and human-made reservoirs may play an important role in shaping zooplankton community structure. For example, because many reservoirs are created by impounding and managing lotic systems for specific human purposes, zooplankton communities may be affected by factors that are unique to reservoirs, such as shorter water residence times and a reservoir’s management regime, compared to natural lakes. However, the environmental factors that structure zooplankton communities in natural lakes vs. reservoirs may vary at the continental scale and remain largely unknown. We analyzed data from the 2007 U.S. Environmental Protection Agency’s National Lakes Assessment and the U.S. Army Corps of Engineers’ National Inventory of Dams to compare large-bodied crustacean zooplankton communities (defined here as individuals retained by 0.243 mm mesh size) in natural lakes and reservoirs across the continental U.S. using multiple linear regressions and regression tree analyses. We found that large-bodied crustacean zooplankton density was overall higher in natural lakes compared to reservoirs when the effect of latitude was controlled. The difference between waterbody types was driven by calanoid copepods, which were also more likely to be dominant in the >0.243 mm zooplankton community in natural lakes than in reservoirs. Regression tree analyses revealed that water residence time was not a major driver of calanoid copepod density in natural lakes but was one of the most important drivers of calanoid copepod density in reservoirs, which had on average 0.5-year shorter water residence times than natural lakes. Reservoirs managed for purposes that resulted in shorter residence times (e.g., hydroelectric power) had lower zooplankton densities than reservoirs managed for purposes that resulted in longer residence times (e.g., irrigation). Consequently, our results indicate that water residence time may be an important characteristic driving differing large-bodied zooplankton dynamics between reservoirs and natural lakes.

## Introduction

Zooplankton are a vital component of aquatic food webs and ecosystem functioning. Zooplankton provide a crucial link between primary producers and higher trophic levels [1–3], are important indicators of ecosystem change [4–7], and can play a key role in lake nutrient and carbon cycling [8–11]. Consequently, differences in zooplankton densities and community composition can have important implications for ecosystem-level processes in lakes, including trophic cascades and water quality [2, 12].

Waterbody origin, i.e., if a waterbody is naturally-formed or human-constructed (a reservoir), may play an important role in structuring zooplankton communities because of inherent differences between the two waterbody types [13–16]. For example, because many reservoirs are constructed by impounding lotic systems [17], they generally have faster flushing rates and shorter water residence times (WRT) than natural lakes [18]. These shorter WRT may result in different zooplankton communities in reservoirs relative to natural lakes. Consequently, reservoirs may have lower zooplankton densities and richness, on average, because zooplankton are continuously washed out of the water column [19–22]. Previous studies on WRT and zooplankton communities, however, have primarily been conducted at a single lake or regional scale. The generality of such relationships at the continental scale, to date, remains untested.

To the best of our knowledge, only two studies have directly examined how zooplankton communities vary between reservoirs and natural lakes [23, 24]. Both studies found that zooplankton community composition differed between the two waterbody types, with some cyclopoid and calanoid copepod taxa occurring more frequently in natural lakes compared to reservoirs [23, 24]. Lower zooplankton species richness in reservoirs and differences in zooplankton composition were attributed to the more eutrophic state, younger geologic age, and possibly greater disturbance of reservoirs compared to natural lakes. However, specific reservoir characteristics such as WRT were not considered in these analyses, and total zooplankton densities and taxa dominance were not directly analyzed. Moreover, the generality of the results may be limited due to the relatively small sample size of reservoirs (11 reservoirs vs. 68 natural lakes) [23], or the focus on waterbodies within only one geographical region (59 waterbodies in southern Brazil) [24].

Differences in reservoir management (here, defined as the primary purpose of a reservoir) may influence zooplankton communities because reservoir purpose may affect many environmental characteristics, such as WRT [22, 23]. For example, reservoirs used mainly for hydroelectric power generation may have shorter WRTs and thereby lower zooplankton densities than reservoirs managed for purposes that result in longer WRTs (e.g., water supply reservoirs) [22, 25]. In addition, reservoir purpose may disproportionately affect the density and richness of certain taxa in the zooplankton community. Because some copepods have long generation times (up to multiple months for an egg to develop into an adult) [1, 26–28], copepods may be more affected by reservoir purpose than cladocerans, which can have generation times of days [1, 26]. A WRT of 1–2 months may negatively affect calanoid copepods, because their generation times may extend up to eight months [1, 26, 28]. Conversely, a WRT of 1–2 months would likely have less of an effect on cladoceran populations, which would still have enough time to grow and reproduce. Thus, copepods may be flushed out of reservoirs with shorter WRT systems compared to longer WRT systems before reaching reproductive adult stages. Because reservoir purpose may alter WRT, which in turn may affect zooplankton density and the dominance of different taxa, reservoir management regime may indirectly affect zooplankton community structure. To the best of our knowledge, such relationships have not yet been tested across reservoirs of multiple primary purposes and at the continental scale.

We analyzed data from the U.S. Environmental Protection Agency’s (EPA) 2007 National Lakes Assessment (NLA) and the U.S. Army Corps of Engineers’ National Inventory of Dams (NID) to test how environmental drivers of crustacean zooplankton density and genera richness (i.e., total crustacean, total copepods, cyclopoid copepods, calanoid copepods, cladocerans, and *Daphnia*) differed between the two waterbody types and across reservoirs of different purposes in the continental U.S. First, we compared zooplankton communities and environmental drivers known to be important factors shaping zooplankton density and composition (e.g., WRT, chlorophyll *a*) between natural lakes and reservoirs at the continental U.S. scale while controlling for the effect of latitude. We predicted that zooplankton density and genera richness would be overall lower in reservoirs than in natural lakes. Second, we focused specifically on reservoirs and analyzed the effects of reservoir primary purpose (e.g., hydropower, recreation) and environmental drivers on zooplankton density. We predicted that zooplankton densities would be lower in reservoirs used for purposes that generally result in shorter WRT (e.g., hydropower) than those used for purposes that result in longer WRT (e.g., recreation).

## Methods

### EPA NLA and sampling

**Fig 1:**
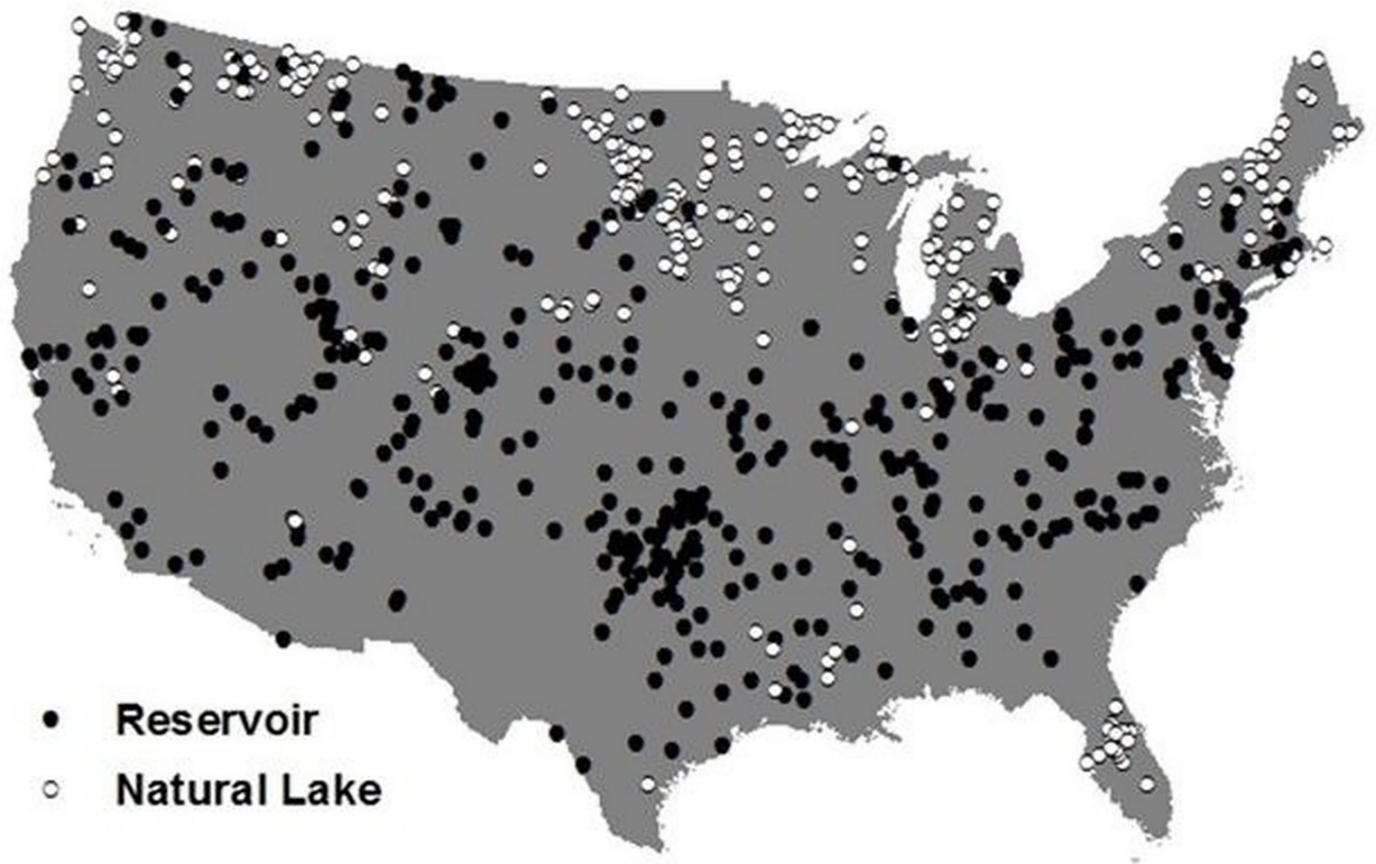
Locations of reservoirs (filled circles) and natural lakes (open circles) sampled in the 2007 National Lakes Assessment that were included in zooplankton analyses.

The NLA sampled both natural lakes and reservoirs across the continental U.S. (Fig 1), providing a large-scale dataset to investigate relationships between the two waterbody types and zooplankton communities. The NLA 2007 data have been used to assess regional to continental patterns of land use, nutrient concentrations, and phytoplankton characteristics [29–34], but have not been used to examine differences in zooplankton density and richness between waterbody types in the U.S.

In 2007, the U.S. EPA sampled >1000 lakes and reservoirs across the U.S. during May to October. Some waterbodies in the NLA had replicate samples or were sampled more than once over the season. We only analyzed data collected on the first sample date to maintain consistency across waterbodies. The mean Julian day when a waterbody was sampled was 212, with a range from day 128 to day 291. No significant relationship existed between the Julian day of sampling and latitude (*P* = 0.73); therefore, there was likely a minimal effect of the time of year when natural lakes were sampled compared to when reservoirs were sampled. All sampled waterbodies were at least 0.04 km^2^ and 1 m deep and were chosen using a random stratified design based on surface area, ecoregion, and state [35, 36]. Reservoir and natural lake designations were included in the NLA dataset and were based on maps, discussions with state and tribal resource managers, and on-site field crew observation. The natural lake category includes waterbodies with water level control devices on naturally-formed basins. Detailed sample collection methods, laboratory processing protocols, and all NLA data are publicly available online (http://water.epa.gov/type/lakes/lakessurvey_index.cfm); thus, we limited our description to the variables included in our analyses.

We analyzed physical, chemical, and biological variables from the NLA dataset known to be important for zooplankton communities to examine potential differences of these variables between reservoirs and natural lakes and their relationships to zooplankton. The variables we included were maximum water column temperature [37–39], mean dissolved oxygen (DO) in the water column [40, 41], pH [42–44], chlorophyll *a* concentrations [45–47], and WRT [22, 48, 49]. Water temperature and DO depth profiles were collected with multi-parameter water quality sondes. Samples for water chemistry and chlorophyll *a* were collected with an integrated tube sampler from the photic zone up to 2 m depth from the surface and sent to a central lab for analysis [29, 32, 33]. WRT was quantified by δ^2^H and δ^18^O ratios from the integrated water sample (see [49] for detailed methods on WRT methods and calculations).

Other variables that may be important for zooplankton communities, such as dissolved organic carbon (DOC) and calcium, were not included because they were highly correlated with the aforementioned environmental variables (pH vs. calcium *r* = 0.74, DOC vs. chlorophyll *a r* = 0.52); other candidate environmental variables that had Pearson product-moment correlations of *r* > 0.50 with the focal environmental variables were excluded from analyses (see S1 Table for Pearson product-moment correlations between environmental variables). We used an *r* of 0.50 as a cut-off to obviate highly correlated variables confounding results in multivariate statistics (see below) [50–52].

### Zooplankton

Zooplankton were collected with vertical net tows (Wildlife Supply Company, Yulee, FL, USA) during daytime on each lake. Natural lakes were sampled at the deepest location in the lake (up to 50 m deep). Reservoirs were sampled at a mid-point in the reservoir (up to 50 m deep). Nets were lowered to 0.5 m above the bottom for both reservoirs and natural lakes and pulled to the surface at a constant speed. A vertical net tow with a 0.243-mm mesh size was used to sample copepod (cyclopoid and calanoid) and cladoceran zooplankton. The plankton net had a 0.127-m diameter opening. All zooplankton were field-preserved with 95% ethanol and sent to a central lab for identification and counting. We acknowledge that the mesh size was larger than some smaller crustacean zooplankton taxa (e.g., *Bosmina* and *Chydorus*), and therefore, much of our focus is on large-bodied crustacean zooplankton which are retained in and minimally affected by a 0.243-mm mesh [1, 26]. We do report results for some zooplankton groups that may be underestimated because of the mesh size, but our conclusions are based primarily on calanoid copepods and *Daphnia*.

Zooplankton were identified using a dissecting microscope. Most taxa were identified to the genus level. For some waterbodies, however, zooplankton were only identified to the order or subclass level (i.e., Cladocera, Calanoida, Cyclopoida, and Copepoda). Counts for cyclopoid and calanoid copepods consisted of copepodids and adults. Copepod nauplii were not considered as they were likely not quantitatively captured with the mesh size. To standardize taxonomic resolution across waterbodies, and because of our interest in analyzing taxa richness, we only included waterbodies that had zooplankton information to the genus level. Most waterbodies in the NLA were still included in our analyses (*N* = 730 waterbodies with zooplankton data).

The NLA reported zooplankton as the number of zooplankton taxa in each subsample, with a minimum of 200 and up to 400 maximum identified individuals. To compare zooplankton data across all waterbodies, we calculated zooplankton density in the water column (individuals L^-1^) by scaling counts per subsample volume to the total volume sampled by the net tows in the water column [1]. Length measurements of zooplankton were not recorded; therefore, we could not calculate zooplankton biomass estimates to quantify potential effects of fish predation on zooplankton communities. More detailed information on zooplankton field and laboratory methods are publicly available online (https://www.epa.gov/sites/production/files/2013-11/documents/2009_12_31_lakes-lakessurvey_pdf_qualityassuranceplan.pdf).

The NLA only reported the presence (but not density) of large predatory zooplankton, such as *Leptodora*, and the insect larvae *Chaoborus*, and thus they were not included in our analyses. Other zooplankton sporadically noted in the dataset (e.g., zebra mussel veligers) were not included due to their rarity (*N* < 20 waterbodies). Fish data were not included in the NLA.

### Statistical analyses

#### Analysis 1: Continental differences in large-bodied zooplankton density, taxa dominance, and genera richness between natural lakes and reservoirs

We first examined major differences in aggregated total density, taxa dominance, and genera richness for each zooplankton group between natural lakes and reservoirs across the continental U.S. The zooplankton groups included in these analyses were: total crustacean zooplankton (the sum of copepods + cladocerans), total copepods (cyclopoids + calanoids), cyclopoids, calanoids, and cladocerans. Total copepods also included harpacticoids, but due to their rarity in these samples (n = 2), they were not analyzed separately. In addition, we performed Analysis 1 for the cladoceran genus *Daphnia*, as an example of a larger-sized cladoceran taxa that should be minimally affected by the size of the zooplankton vertical tow nets [1]. The dominance of each taxon group in the crustacean zooplankton community was calculated by dividing the density of the group (total copepods, cyclopoids, calanoids, and cladocerans) by the total crustacean zooplankton density for each waterbody.

Many physical, chemical, and biological factors vary with latitude [53–55]. Consequently, we also accounted for latitude as a covariate in waterbody origin comparisons because of the large geographic disparity in the location of natural lakes (dominant in northern latitudes) vs. reservoirs (dominant in southern latitudes) in the U.S. The effects of waterbody type (natural lakes or reservoirs), latitude, and their interaction were analyzed on our response variables using multiple linear regression models with waterbody type as an indicator variable (coded as natural lakes as 0 and reservoirs as 1) [56]. The regression equation we used for each analysis was:

$$\text{Y}={\text{B}}_{0}+{\text{B}}_{1}{\text{X}}_{\text{waterbody}\_\text{type}}+{\text{B}}_{2}{\text{X}}_{\text{latitude}}+{\text{B}}_{3}{\text{X}}_{\text{waterbody}\_\text{type}\times \text{latitude}}+\varepsilon$$ (1)

where Y is the response variable of interest, B_0_ is the intercept term, B_1_, B_2_, and B_3_ are the respective model coefficient terms, and ɛ is the stochastic error term.

At the continental scale, we also tested the effects of waterbody type, latitude, and their interaction on maximum water column temperature, mean water column DO, pH, chlorophyll *a*, and WRT (environmental variables that are known to be important for zooplankton community structure) in multiple linear regressions, as in the above analyses for zooplankton. Because each zooplankton response variable and other environmental response variables were only used once in statistical analyses, and hence were in separate families of tests, correction of *P*-values was not necessary [56, 57].

#### Analysis 2: Regression tree analyses of the effects of environmental factors on large-bodied zooplankton densities

Analysis 1 tested for broad-scale differences in aggregated total density, taxa dominance, and genera richness between waterbody types across the U.S. For Analysis 2, we expanded on Analysis 1 by examining which environmental variables may contribute to differences in zooplankton densities between reservoirs and natural lakes. We performed regression tree analyses to compare the relative importance of the focal environmental factors on crustacean zooplankton, total copepod, calanoid, cladoceran, and *Daphnia* density between reservoirs and natural lakes. We did not include latitude in regression tree analyses because our goal was to assess the impacts of direct environmental variables (e.g., maximum water temperature, chlorophyll *a*, and WRT) on zooplankton communities and because latitude is highly correlated with many environmental variables in our analysis that have direct effects on zooplankton densities. However, we examined any latitudinal or regional clustering from the regression tree results by plotting the geographic location of crustacean zooplankton density groupings based upon the splits in the regression tree analysis.

Regression trees provide a robust statistical approach to handle potential non-linear relationships and nested effects among variables [58–60]. Pruned regression trees were chosen by minimizing cross-validation error [60, 61]. We performed regression trees for crustacean, total copepod, calanoid, cladoceran, and *Daphnia* density in all waterbodies together and then separately for natural lakes only and reservoirs only, focusing on the waterbodies that had all focal environmental variables available (*N* = 688 waterbodies). Regression tree analyses were performed using the R package “rpart” in R v3.2.4 [62].

#### Analysis 3: Relationships between reservoir primary purpose, environmental factors, and large-bodied zooplankton densities

Next, we examined how differences among reservoirs with varying primary purpose and environmental factors may affect crustacean zooplankton community structure. The NID (http://nid.usace.army.mil/) designates and defines the primary purpose of each reservoir in the U.S. as hydropower, recreation, water supply, irrigation, or flood control (*N* > 20 for each of these categories). We used the NID classifications to assess differences among primary purpose and maximum water column temperature, mean water column DO, pH, chlorophyll *a* concentrations, WRT, total crustacean, total copepod, cyclopoid, calanoid, cladoceran, and *Daphnia* density using one-way ANOVA with post-hoc Tukey pairwise comparisons. For the reservoir-only analyses, we included all the reservoirs in the NLA for which the NID also had available data on primary purpose (*N* = 303). In contrast to the reservoir vs. natural lakes comparison in Analysis 1, the use of ANOVA was appropriate for the reservoir-only analyses because reservoirs with different purposes were geographically distributed across diverse regions in the U.S., without a latitudinal bias as for all zooplankton response variables (S1 Fig contains the location of the five reservoir primary purpose types).

To meet assumptions of normality and equal variance, total crustacean zooplankton density, total copepod density, cyclopoid density, calanoid density, cladoceran density, *Daphnia* density, chlorophyll *a*, and WRT were ln-transformed for all statistical analyses. Variables expressed as proportions (total copepod, cyclopoid, calanoid, and cladoceran dominance) were logit-transformed prior to analyses [63]. If variables had zero values, the minimum observed value for each variable was added prior to transformation. The *P*-values for statistical tests were considered significant at α ≤ 0.05. All analyses were performed in R v3.2.4 [62].

## Results

### Analysis 1: Continental differences in large-bodied zooplankton density, taxa dominance, and genera richness between natural lakes and reservoirs

**Fig 2:**
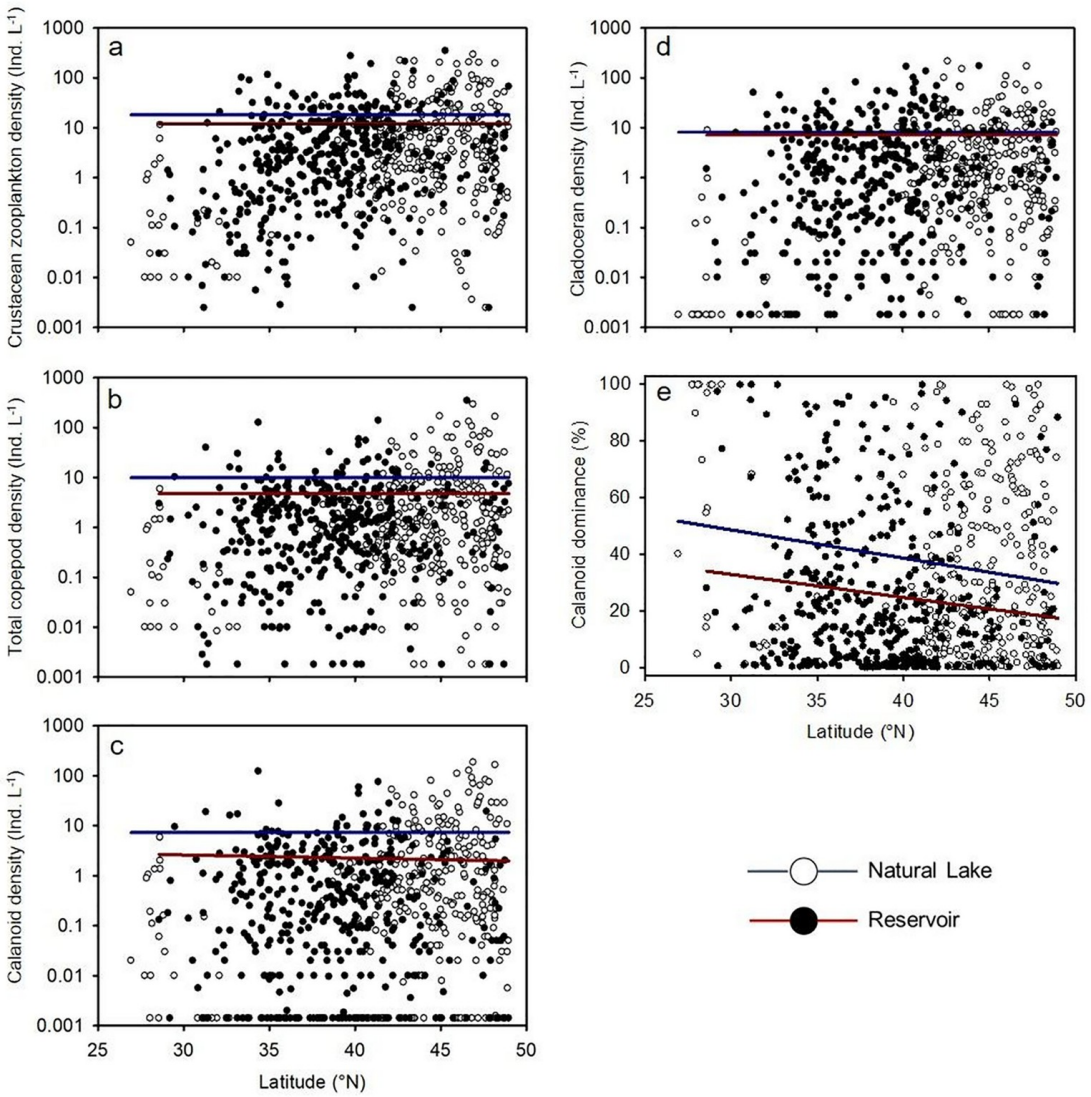
U.S.-scale comparisons of a) crustacean zooplankton (copepod + cladoceran), b) total copepod (cyclopoid + calanoid), c) calanoid, and d) cladoceran density, and e) calanoid dominance in the zooplankton community between natural lakes and reservoirs and across latitude. Note that the y-axis is in log scale for the zooplankton density panels.

Natural lakes across the U.S. had higher total crustacean zooplankton density than reservoirs (*F*_3,726_ = 5.26; *P* = 0.02; Fig 2A) when also accounting for latitude. Natural lakes had an untransformed mean density (± 1 SE) of 18 ± 2 individuals L^-1^ compared to reservoirs which had 12 ± 1 individuals L^-1^. The difference in crustacean zooplankton density was driven primarily by more than 3× higher densities of calanoid copepods in natural lakes versus reservoirs, which resulted in more than 2× higher densities of total copepods in natural lakes (*F*_3,726_ ≥ 8.36; *P* ≤ 0.004; Tables 1 and 2; Fig 2B and 2C; S2 Fig). In contrast to calanoids, cladoceran (Fig 2D), *Daphnia*, and cyclopoid copepod densities did not differ significantly between waterbody types (*P* ≥ 0.40). No interaction existed between waterbody type and latitude for any zooplankton density response variable (all *P* ≥ 0.13).

**Table 1:** Comparison of each zooplankton group and other environmental variables between natural lakes and reservoirs across the continental U.S., with each variable’s untransformed mean values and standard errors (SE).

Variable	Natural Lakes Mean ± SE	Reservoirs Mean ± SE
**Zooplankton response variables**		
Total crustacean density*	18.1 ± 2.3 L^-1^	11.8 ± 1.5 L^-1^
Total copepod density*	9.9 ± 1.6 L^-1^	4.7 ± 1.0 L^-1^
Cyclopoid density	2.6 ± 0.7 L^-1^	2.4 ± 0.8 L^-1^
Calanoid density*	7.3 ± 1.2 L^-1^	2.3 ± 0.4 L^-1^
Cladoceran density	8.1 ± 1.3 L^-1^	7.1 ± 0.9 L^-1^
*Daphnia* density	3.4 ± 0.5 L^-1^	3.3 ± 0.5 L^-1^
Total copepod dominance*	54.0 ± 1.8%	49.0 ± 1.4%
Cyclopoid dominance*	19.2 ± 1.4%	23.5 ± 1.2%
Calanoid dominance*	35.1 ± 1.8%	25.8 ± 1.3%
Cladoceran dominance*	46.8 ± 1.7%	52.0 ± 1.4%
Total crustacean zooplankton genera richness	4.15 ± 0.09	4.08 ± 0.07
Total copepod genera richness	1.94 ± 0.04	1.86 ± 0.03
Cyclopoid genera richness	0.94 ± 0.02	0.96 ± 0.02
Calanoid genera richness*	1.01 ± 0.03	0.90 ± 0.02
Cladoceran genera richness	2.21 ± 0.07	2.22 ± 0.05
**Other environmental variables**		
Maximum water column temperature*	22.9 ± 0.2 °C	24.7 ± 0.2 °C
Mean water column dissolved oxygen*	6.4 ± 0.1 mg L^-1^	6.0 ± 0.1 mg L^-1^
pH	8.1 ± 0.05	8.0 ± 0.03
Chlorophyll *a*	38.6 ± 5.1 μg L^-1^	23.6 ± 2.3 μg L^-1^
Water residence time*	1.3 ± 0.1 years	0.8 ± 0.1 years

The response variables that were significantly different between natural lakes and reservoirs when also accounting for latitude in models are highlighted with an asterisk (*). All statistics for the natural lakes vs. reservoirs comparison are given in Table 2.

**Table 2:** Multiple linear regression model statistics for the effects of waterbody type as an indicator variable (with natural lakes coded as 0 and reservoirs coded as 1), latitude, and the interaction of waterbody type and latitude on zooplankton and other environmental variables.

Response Variable	*n*_lakes_	*n*_reservoirs_	Intercept parameter ± SE	Waterbody Type		Latitude		Interaction	
				Value ± SE	*P*	Value ± SE	*P*	Value ± SE	*P*
**Zooplankton**									
ln(crustacean zooplankton density)	301	429	-6.57 ± 1.12	1.69 ± 1.48	**0.02**	0.18 ± 0.03	**<0.0001**	-0.03 ± 0.04	0.37
ln(total copepod density)	301	429	-6.21 ± 1.16	1.57 ± 1.53	**0.004**	0.15 ± 0.03	**<0.0001**	-0.03 ± 0.04	0.34
ln(cyclopoid density)	301	429	-9.32 ± 1.46	3.68 ± 1.92	0.47	0.17 ± 0.03	**<0.0001**	-0.07 ± 0.05	0.13
ln(calanoid density)	301	429	-7.21 ± 1.50	2.48 ± 1.98	**<0.0001**	0.15 ± 0.03	**<0.0001**	-0.07 ± 0.05	0.14
ln(cladoceran density)	301	429	-10.37 ± 1.36	2.87 ± 1.79	0.40	0.23 ± 0.03	**<0.0001**	-0.05 ± 0.04	0.25
ln(*Daphnia* density)	301	429	-12.72 ± 1.55	1.90 ± 2.04	0.74	0.26 ± 0.04	**<0.0001**	-0.02 ± 0.05	0.69
logit(total copepod dominance)	301	429	3.57 ± 0.50	-1.84 ± 0.66	**0.03**	-0.08 ± 0.01	**<0.0001**	0.03 ± 0.02	**0.03**
logit(cyclopoid dominance)	301	429	-0.78 ± 0.57	0.70 ± 0.76	**0.002**	-0.01 ± 0.01	0.12	-0.01 ± 0.02	0.50
logit(calanoid dominance)	301	429	1.74 ± 0.61	-1.01 ± 0.80	**<0.0001**	-0.05 ± 0.01	**<0.0001**	0.01 ± 0.02	0.60
logit(cladoceran dominance)	301	429	-1.76 ± 0.46	0.70 ± 0.61	**0.02**	0.04 ± 0.01	**<0.0001**	-0.01 ± 0.01	0.52
Total crustacean zooplankton genera richness	301	429	-0.44 ± 0.73	4.23 ± 0.96	0.59	0.11 ± 0.02	**<0.0001**	-0.10 ± 0.02	**<0.0001**
Total copepod genera richness	301	429	0.66 ± 0.33	1.33 ± 0.43	0.10	0.03 ± 0.01	**0.02**	-0.03 ± 0.01	**0.002**
Cyclopoid genera richness	301	429	0.45 ± 0.21	0.54 ± 0.27	0.43	0.01 ± 0.005	0.13	-0.01 ± 0.01	0.07
Calanoid genera richness	301	429	0.21 ± 0.24	0.79 ± 0.32	**0.004**	0.02 ± 0.01	**0.05**	-0.02 ± 0.01	**0.007**
Cladoceran genera richness	301	429	-1.01 ± 0.58	2.56 ± 0.76	0.86	0.07 ± 0.01	**<0.0001**	-0.06 ± 0.02	**0.002**
**Other environmental variables**									
Maximum water column temperature	300	425	41.52 ± 1.86	3.04 ± 2.44	**<0.0001**	-0.43 ± 0.04	**<0.0001**	-0.09 ± 0.06	0.15
Mean water column dissolved oxygen	298	394	4.29 ± 1.07	-3.46 ± 1.42	**0.01**	0.05 ± 0.02	**<0.0001**	0.08 ± 0.03	**0.01**
pH	301	429	7.61 ± 0.38	-0.72 ± 0.49	0.22	0.01 ± 0.01	**0.0006**	0.02 ± 0.01	0.13
ln(chlorophyll *a*)	300	426	6.02 ± 0.79	-1.19 ± 1.04	0.81	-0.09 ± 0.02	**<0.0001**	0.02 ± 0.02	0.41
ln(water residence time)	301	429	-2.31 ± 0.61	0.69 ± 0.81	**<0.0001**	0.05 ± 0.01	**0.001**	-0.03 ± 0.02	0.14

Predictor term parameters with their standard error (SE) are provided for each model component. Statistically significant results are highlighted in bold and *n* is the sample size of waterbodies in each analysis. ln() and logit() denote ln-transformations and logit-transformations of certain response variables, respectively.

Copepods, predominantly calanoids, dominated the large-bodied crustacean zooplankton community in natural lakes (defined by the proportion of each taxon in the crustacean zooplankton community for each waterbody; *F*_3,726_ ≥ 4.62; *P* ≤ 0.03; Fig 2E), whereas cladocerans dominated in reservoirs (*F*_3,726_ = 5.96; *P* = 0.01). On average, total copepods composed ~5% more, and calanoids composed ~10% more, of the crustacean zooplankton community in natural lakes than in reservoirs (Tables 1 and 2; S2 Fig). The interaction of waterbody type and latitude was significant for the dominance of copepods in the zooplankton community (*P* = 0.03); the differences in copepod dominance between waterbody types became similar at higher latitudes between natural lakes and reservoirs.

Calanoids had higher genera richness in natural lakes than in reservoirs (*F*_3,726_ = 2.49; *P* = 0.01); however, no difference existed in crustacean zooplankton, copepod, cladoceran, or cyclopoid genera richness between waterbody types (*P* ≥ 0.10; Table 1). A significant waterbody and latitude interaction was also present for calanoid genera richness (*P* = 0.007): calanoids had higher genera richness in natural lakes vs. reservoirs at higher latitudes (>38 °N) and higher genera richness in reservoirs vs. natural lakes at lower latitudes. Although differences in genera richness of calanoids between natural lakes and reservoirs were relatively small, natural lakes were 2× more likely to have at least two calanoid genera present compared to reservoirs (Tables 1 and 2; S2 Fig).

Reservoirs, on average, had about half the WRT compared to natural lakes (*F*_3,729_ = 52.10; *P* < 0.0001; 0.8 ± 0.1 years vs. 1.3 ± 0.1 years) and were 2°C warmer than natural lakes across the continental U.S, while controlling for latitude (*F*_3,724_ = 44.52; *P* < 0.001; 24.7 ± 0.2 °C vs. 22.9 ± 0.2 °C, respectively; Tables 1 and 2). Although not significantly different, reservoirs had, on average, one-half of the chlorophyll *a* concentration of natural lakes (*P* = 0.81; 23.6 ± 2.3 μg/L vs. 38.6 ± 5.1 μg/L). A significant waterbody type and latitude interaction existed for mean DO concentrations (*P* = 0.03); reservoirs had greater mean DO concentrations than natural lakes at higher latitudes (>40 °N) and lower mean DO concentrations than natural lakes at lower latitudes (Tables 1 and 2). pH values were similar between waterbody types across the U.S (*P* = 0.22).

### Analysis 2: Regression tree analyses of the effects of environmental factors on large-bodied zooplankton densities

**Fig 3:**
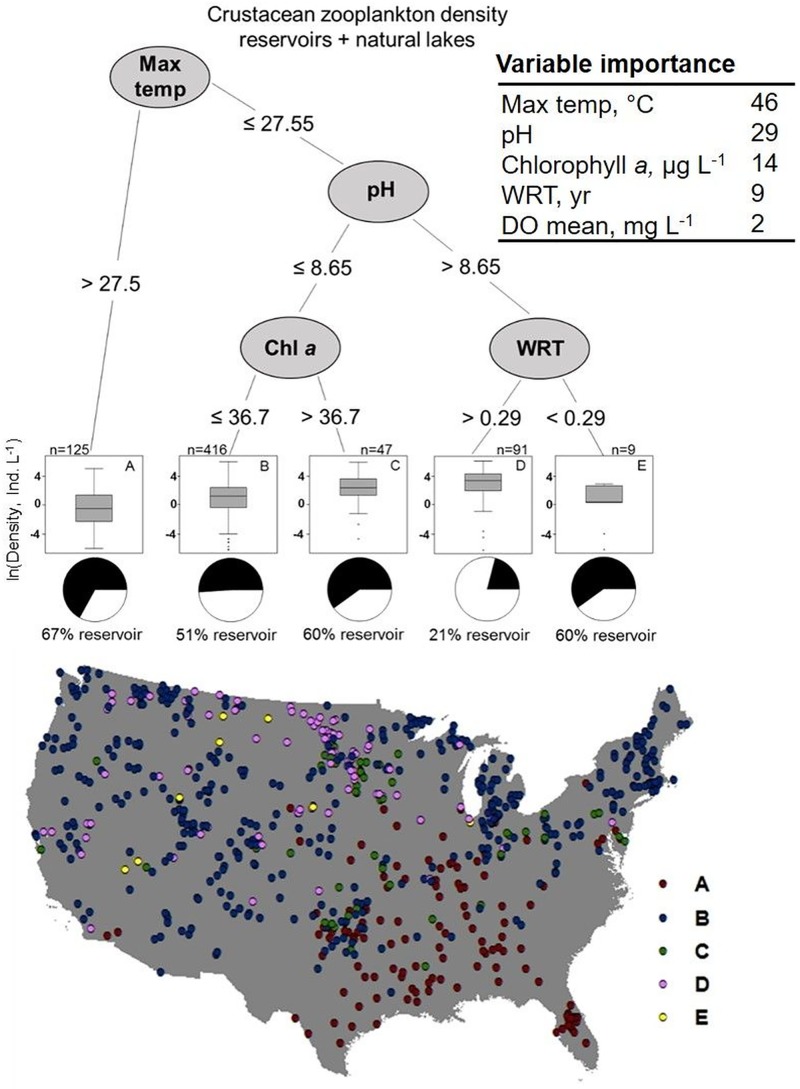
Regression tree analysis for crustacean zooplankton densities aggregated across all waterbodies in the U.S., with the locations of the waterbodies from the regression tree groupings shown with different colors on the map. In the pie charts, white refers to natural lakes and black refers to reservoirs.

Maximum water column temperature and pH were the most important focal environmental variables affecting crustacean zooplankton density when both waterbody types were aggregated across the U.S. (Fig 3; S2 and S3 Tables provide all the regression tree statistics for all response variables for all waterbodies, reservoirs only, and natural lakes only). The highest crustacean zooplankton densities were found in waterbodies that had less than 27.5 °C maximum water column temperature (the first split in the regression tree), greater than 8.65 pH, and longer WRT (> 0.29 years; Group D in Fig 3). Crustacean zooplankton in these waterbodies had a mean density of 48.7 ± 6.7 zooplankton L^-1^, which was approximately 2× higher than the mean zooplankton density in waterbodies with lower pH, but higher chlorophyll *a* concentrations (29.2 ± 7.0 zooplankton L^-1^, Group C in Fig 3). Waterbodies that had the longest WRT and highest crustacean zooplankton densities were predominantly natural lakes (79%) and were spread throughout many regions in the U.S (Group D in Fig 3). Of all waterbodies, those with shorter WRT, despite lower temperatures and higher pH concentrations, had the lowest mean density of crustacean zooplankton (6.0 ± 2.1 zooplankton L^-1^, Group E). Mean water column DO concentration was the least important focal environmental variable in explaining crustacean zooplankton densities across all waterbodies.

**Fig 4:**
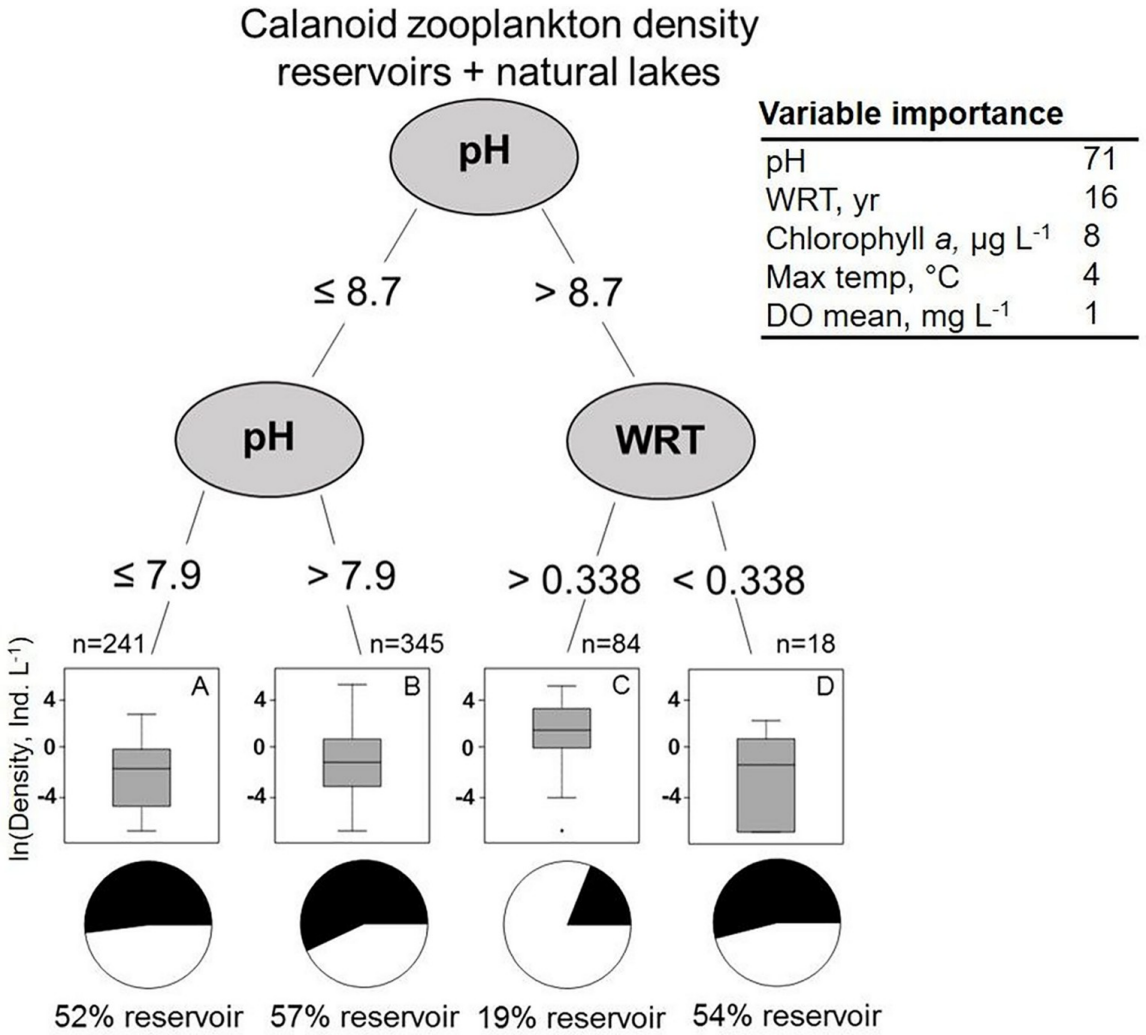
Regression tree analysis for calanoid zooplankton density aggregated across all waterbodies (reservoirs and natural lakes together) in the U.S. In the pie charts, white refers to natural lakes and black refers to reservoirs.

Across all aggregated waterbodies, pH was the first split in the regression tree for calanoid density and thus the most important of the focal environmental variables, with WRT as the second most important variable (Fig 4). Mean calanoid density was highest in waterbodies with higher pH (> 8.7) and longer WRT (> 0.34 yrs; Group D, Fig 4). Regardless of pH, waterbodies with shorter WRT had approximately 9× lower calanoid densities than waterbodies with longer WRT (2.6 ± 0.9 vs. 18.7 ± 3.3 calanoids L^-1^, respectively). Only 19% of the waterbodies with longer WRT and higher pH were reservoirs (Group C in Fig 4), whereas reservoirs composed 54% of the shorter WRT waterbodies in Group D. Waterbodies across the regression tree splits for calanoid densities were generally located across multiple regions in the U.S. (S3 Fig).

**Fig 5:**
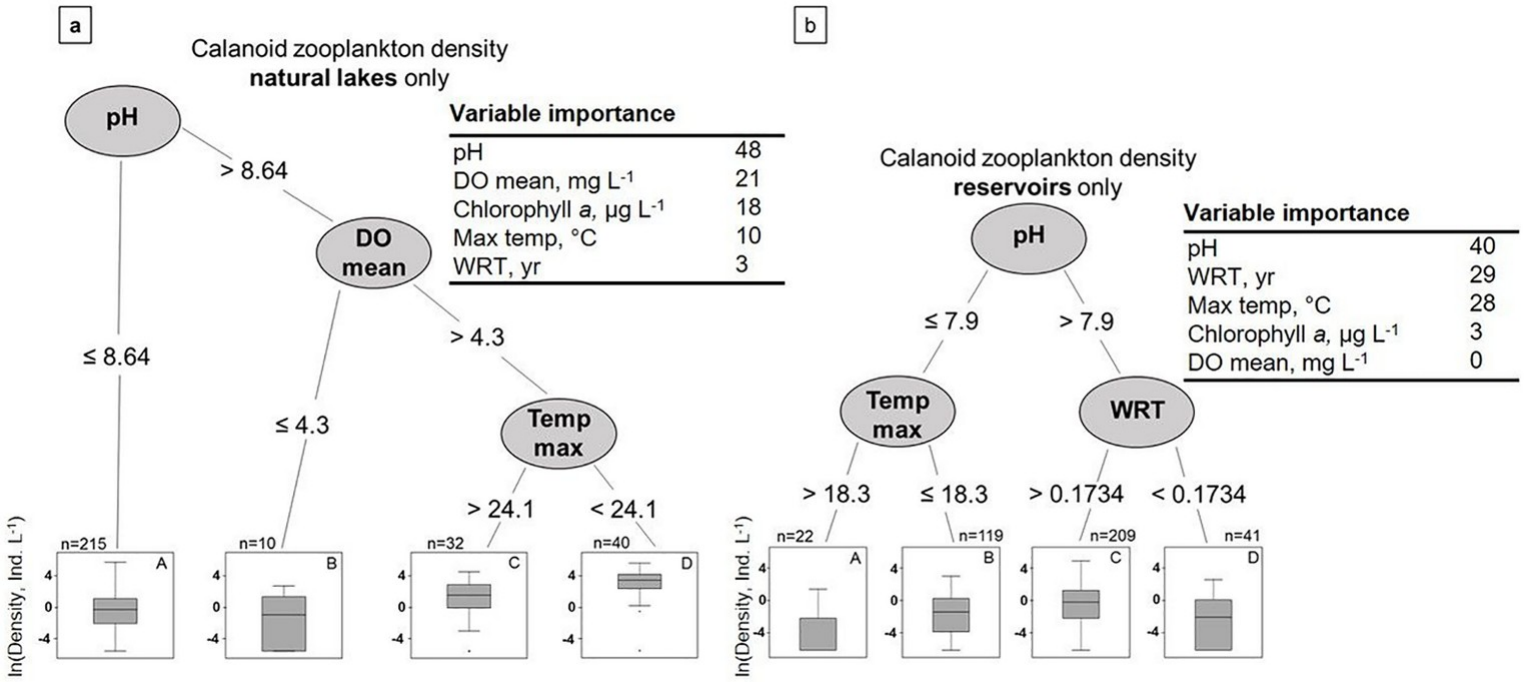
Regression tree analysis for calanoid zooplankton density conducted separately for a) natural lakes and b) reservoirs.

The separate regression trees for reservoirs and natural lakes highlight that different factors were responsible for driving calanoid density in the two waterbody types (Figs 4 and 5). Specifically, WRT was about 10× more important for calanoid copepods in reservoirs compared to natural lakes likely due to overall shorter WRT in reservoirs (Fig 5; S2 and S3 Tables). In natural lakes, pH, DO, and chlorophyll *a* concentrations were the most important environmental variables for calanoid density, whereas WRT was the least important variable. In comparison, in reservoirs, WRT was the second most important variable behind pH, contributing a similar amount of variance to calanoid copepod density as maximum waterbody temperature. In reservoirs, the highest calanoid densities were in waterbodies that had higher pH (> 7.9) and longer WRT (> 0.17 years; 3.8 ± 0.8 calanoids L^-1^, Group C in Fig 5B). Despite having higher pH, calanoids in shorter WRT reservoirs had approximately 4× lower densities than calanoids in longer WRT reservoirs (1.1 ± 0.4 calanoids L^-1^; Fig 5B, Groups C and D). WRT in natural lakes and reservoirs was not as important for cyclopoid or cladoceran density compared to calanoids (S2 and S3 Tables). Regression tree results for *Daphnia* density were similar to results for cladoceran density in all waterbodies, in just natural lakes, and in just reservoirs (S2 and S3 Tables); pH and maximum water column temperature were the most important environmental factors for *Daphnia* density.

Chemical and biological variables (DO, chlorophyll *a*, and pH) were overall more important for zooplankton groups in natural lakes, whereas physical variables were more important for zooplankton in reservoirs (maximum water temperature and WRT; Fig 5; S3 Table).

### Analysis 3: Relationships between reservoir primary purpose, environmental factors, and large-bodied zooplankton densities

**Fig 6:**
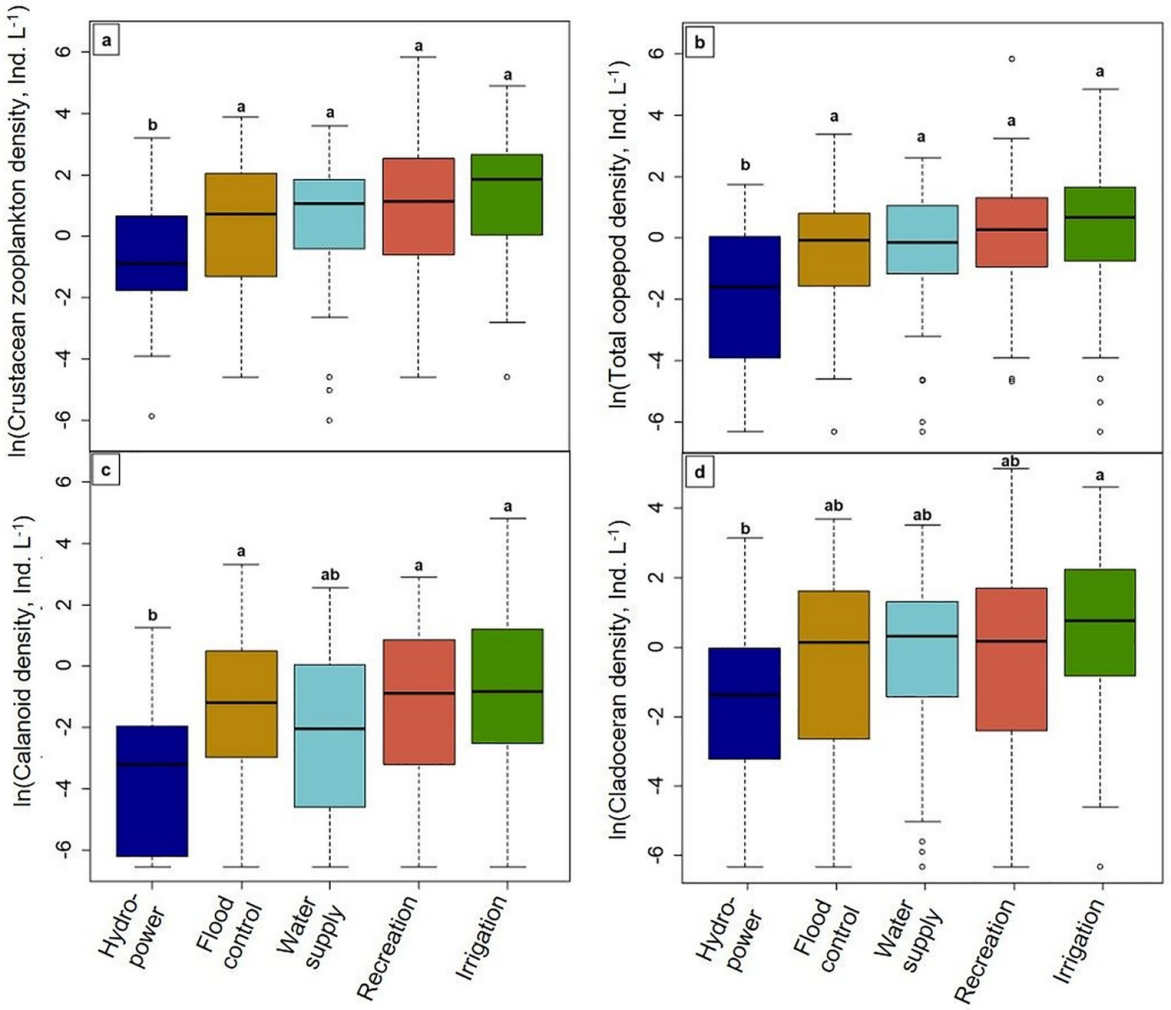
Relationships between reservoir primary purpose and a) total crustacean zooplankton, b) total copepods, c) calanoids, and d) cladocerans. Letters above each boxplot denote statistical differences of zooplankton among reservoir primary purpose.

Total crustacean zooplankton, total copepod, calanoid, and cladoceran densities were significantly different across reservoirs used for different primary purposes (*F*_4,298_ ≥ 3.43; *P* ≤ 0.009; Fig 6). On average, total crustacean zooplankton densities were about 4× lower in hydroelectric reservoirs (mean 3.1 ± 1.1 zooplankton L^-1^) than in reservoirs primarily used for irrigation (14.0 ± 3.0 zooplankton L^-1^; P < 0.0001) or recreation (14.2 ± 4.8 zooplankton L^-1^; *P* = 0.001), and half as low as in reservoirs primarily used for flood control and water supply (7.7 ± 1.6 and 5.9 ± 1.0 zooplankton L^-1^, respectively; *P* ≤ 0.03; Fig 6A). Total copepod (Fig 6B) and calanoid densities (Fig 6C) were about an order of magnitude lower in hydroelectric reservoirs (0.8 ± 0.2 and 0.4 ± 0.1 individuals L^-1^, respectively) than reservoirs primarily used for irrigation (5.7 ± 2.0 copepods L^-1^ and 4.0 ± 1.9 calanoids L^-1^) and recreation (7.1 ± 4.2 copepods L^-1^ and 1.6 ± 0.3 calanoids L^-1^; all *P* ≤ 0.007). Cladoceran density in hydroelectric reservoirs was only significantly lower than in reservoirs used for irrigation (Fig 6D), and *Daphnia* density was only significantly lower in reservoirs used for recreation than reservoirs used for irrigation (*F*_4,298_ = 3.11; *P* = 0.02).

WRT varied significantly across reservoirs with different primary purposes (*F*_4,298_ = 5.32, *P* = 0.0004; Table 3). WRT was approximately 2× shorter in reservoirs used for hydroelectric power, flood control, or recreation than reservoirs used primarily for irrigation (all *P* ≤ 0.008). Water supply reservoirs had intermediate WRT (Table 3). Maximum water column temperature was significantly different across reservoirs with different purposes (*F*_4,295_ = 15.35; *P* < 0.0001; Table 3), with reservoirs used primarily for irrigation having lower temperatures than reservoirs used for recreation, flood control, or water supply (all *P* ≤ 0.01). Chlorophyll *a* was also significantly lower in reservoirs primarily used for irrigation or hydroelectric power than reservoirs used for recreation (*F*_4,296_ = 4.47; *P* = 0.002). Reservoirs used for irrigation had a greater mean water column DO than reservoirs used primarily for recreation or flood control (*F*_4,269_ = 3.73; *P* = 0.006). No significant difference in pH across reservoir purposes was observed (*P* = 0.24).

**Table 3:** Mean values (± 1 SE) for the focal environmental variables across reservoirs of different primary purposes, ordered by their WRT.

Primary purpose	*N*	WRT (years)	Maximum water temperature (°C)	Mean DO (mg L^-1^)	pH	Chlorophyll *a* (μg L^-1^)
Recreation	83	0.50 ± 0.05 (b)	26.1 ± 0.4 (a)	5.6 ± 0.3 (b)	7.9 ± 0.09 (a)	37.8 ± 8.0 (a)
Flood control	67	0.55 ± 0.07 (b)	25.8 ± 0.3 (ab)	5.5 ± 0.2 (b)	8.0 ± 0.06 (a)	20.1 ± 3.4 (ab)
Hydropower	31	0.64 ± 0.13 (b)	23.5 ± 1.0 (bc)	6.2 ± 0.4 (ab)	7.9 ± 0.09 (a)	10.8 ± 2.5 (b)
Water supply	58	0.70 ± 0.10 (ab)	24.7 ± 0.7 (ab)	5.9 ± 0.3 (ab)	7.9 ± 0.08 (a)	15.8 ± 2.7 (ab)
Irrigation	64	1.2 ± 0.17 (a)	21.5 ± 0.4 (c)	6.8 ± 0.3 (a)	8.1 ± 0.07 (a)	20.1 ± 6.2 (b)

Letters denote statistical differences across different reservoir purposes based on one-way ANOVA with post-hoc Tukey pairwise comparisons. *N* refers to the sample size of each reservoir purpose.

## Discussion

While many studies have quantified how many physical, chemical, and biological factors affect zooplankton community structure, results from our study highlight the role of waterbody type in driving large-bodied crustacean zooplankton (retained in mesh size > 0.243 mm) communities across the continental U.S. Overall, we found natural lakes to have significantly greater density and dominance by copepods, especially calanoid copepods. Calanoids were also more likely to have multiple genera present in natural lakes compared to reservoirs.

Calanoid copepods were responsible for most crustacean zooplankton density differences between waterbody types when also accounting for latitude. Shorter WRT in reservoirs compared to natural lakes may be a major factor explaining waterbody type differences in calanoid communities [20, 22, 48]. According to the regression tree analysis, we observed the highest calanoid and crustacean zooplankton densities in waterbodies that had longer WRT and higher pH, of which most (~80%) were natural lakes (Figs 3 and 4). In contrast, calanoid and total crustacean zooplankton densities were about 3 and 9× lower, respectively, in waterbodies that also had high pH, but shorter WRT. Many calanoid taxa may be particularly influenced by shorter WRT because individuals within these groups take longer to reach reproductive maturity, and exhibit fewer seasonal peaks in the density of egg-bearing females than cyclopoid copepods and cladocerans during a summer season [1, 26, 27].

The significance of WRT and calanoid copepod density is highlighted by the WRT values in the regression tree analyses (0.17 to 0.34 years) that result in decreased calanoid copepod density. Calanoid copepod taxa exhibit a large range in the duration of time for individuals to reach reproductive maturity. For example, smaller calanoids such as *Diaptomus* and *Epischura* can reach reproductive maturity within a few months, whereas larger calanoids such as *Limnocalanus* and *Senecella* can take 6–8 months [28, 64, 65]. Therefore, the exact threshold value of WRT (0.17 years in reservoirs, or ~2 months) may be a signal of the overall calanoid community composition in the U.S. Conversely, shorter WRT does not appear to have much effect on faster-growing Cladocera, such as *Daphnia*, which can grow and reach reproductive maturity within days [26]. Shorter WRT can also affect many other waterbody environmental conditions, such as chlorophyll *a* concentrations, which may indirectly affect zooplankton (in addition to the direct effect of physically flushing zooplankton out of a waterbody). However, previous studies have found that WRT has little effect on overall primary productivity because chlorophyll *a* is able to increase quickly after disturbance [48, 66]. Therefore, the direct and indirect effects of WRT are likely to negatively influence calanoid copepods more than cladocerans because cladocerans are overall more herbivorous than calanoids.

Other factors, such as chlorophyll *a*, likely play a role in zooplankton community differences among waterbody types [45, 67]. Calanoids often dominate zooplankton communities in more oligotrophic systems [68–70]; however, reservoirs were more oligotrophic than natural lakes in the 2007 NLA dataset (Table 1), indicating that other environmental factors, such as shorter WRT, may be more important in driving the calanoid community than chlorophyll *a* concentrations in reservoirs.

Crustacean zooplankton density across all waterbodies was most strongly affected by maximum waterbody temperature, with decreased densities when maximum waterbody temperatures exceeded 27.5°C, likely because temperatures reaching 25–30°C are detrimental to crustacean zooplankton growth and survival in temperate waterbodies [71, 72]. Many high maximum temperature waterbodies were in warmer regions in the U.S., such as the Southeast and Central Plains, and two-thirds of these waterbodies were reservoirs (Fig 3). Other studies have observed decreased zooplankton densities in waterbodies in warmer regions in the U.S., predominantly of cladocerans [38, 73]. Cladocerans, exemplified by *Daphnia*, were most affected by maximum water column temperature compared to other zooplankton taxa (S2 Table; S4 Fig), indicating a possible lower thermal tolerance for cladocerans compared to copepods in temperate waterbodies [74]. Hence, higher water temperatures may be another reason for lower overall crustacean zooplankton densities in reservoirs, which is likely a result of the latitudinal distribution of natural lakes and reservoirs across the U.S. (Fig 1). In addition, pH was often an important variable for zooplankton density across analyses. Crustacean zooplankton densities were lower in waterbodies with pH less than ~7.8–8.7; as pH decreases, zooplankton growth and survival is constrained by increased stress to lower pH values, which overall lowers the density of zooplankton [75–77].

Maximum water column temperature or waterbody depth differences between reservoirs and natural lakes may have contributed to the lower number of calanoid genera generally present in reservoirs compared to natural lakes. Waterbody maximum depth is positively related to species richness because deeper waterbodies have more niches [78–80]; however, maximum waterbody depth was similar between natural lakes and reservoirs in the NLA (10.5 ± 13.0 m (1 S.D.) and 9.7 ± 9.9 m, respectively). Waterbody age differences between reservoirs and natural lakes may have also played a role in the richness differences. Younger, more geographically isolated reservoirs may have experienced lower rates of zooplankton dispersal, and thus fewer sexual-reproducing calanoid genera present than older natural lakes [27]. However, previous studies have found no significant relationship between reservoir age and zooplankton richness [23, 81], and we were not able to quantify the effect of age versus other environmental factors on calanoid genera richness with the 2007 NLA data.

The relative importance of environmental factors on zooplankton densities differed between natural lakes and reservoirs. In general, the focal physical variables (maximum water column temperature and WRT) were more important for zooplankton densities in reservoirs compared to natural lakes, whereas the focal chemical and biological factors were overall more important for zooplankton in natural lakes. Reservoirs are generally more common in warmer regions in the U.S. (Fig 1) [14, 82], and those constructed by damming lotic ecosystems are more greatly affected by WRT than natural lakes [20, 22, 48]; therefore, the greater relative importance of physical variables for zooplankton in reservoirs is not surprising. In contrast, because natural lakes generally have lower discharge rates and longer WRT [18], physical variables in natural lakes may be less important in structuring zooplankton communities compared to reservoirs. Subsequently, chemical and biological factors may be more important overall for zooplankton communities in natural lakes at single snapshots in time.

Reservoir WRT was significantly different across reservoirs of different primary purposes. Recreation, hydroelectric, and flood control reservoirs had half the WRT, on average, of reservoirs used for irrigation, which likely is related to differences in reservoir management operations. For example, hydroelectric reservoirs may have shorter WRT to meet electrical power demands [83, 84], and flood control reservoirs may have shorter WRT because of the need to quickly store and release water levels in response to weather [85, 86]. Reservoirs used primarily for irrigation or water supply may be more likely, on average, to have longer WRT, because of the importance to maintain water levels for drinking water and irrigation.

Our results suggest that zooplankton densities differed between reservoirs of different primary purposes as a result of differences in WRT: hydroelectric reservoirs (with shorter WRT) had the lowest zooplankton densities and irrigation reservoirs (with longer WRT) had the greatest zooplankton densities. Total copepod and calanoid zooplankton densities were especially lower in hydroelectric reservoirs versus most other reservoir purposes (Fig 6), with densities typically <1 individual L^-1^. High discharge reservoir purposes, such as hydroelectric reservoirs, may flush many of these taxa from the system before they become adults. Other factors certainly also play a role in these density differences across reservoirs of different purposes, as many other environmental factors varied across reservoir purposes. For example, chlorophyll *a* was lower in hydroelectric reservoirs versus some other reservoir types, which can result in lower zooplankton densities [2, 38, 66]. However, chlorophyll *a* was also lower in reservoirs used for irrigation, which had the greatest crustacean zooplankton densities. Overall, our results suggested that reservoir purpose likely affects multiple environmental factors, which in turn can indirectly alter zooplankton densities and community structure.

The EPA’s NLA had excellent spatial coverage of waterbodies. However, one limitation of our work is that most lakes and reservoirs were sampled only once and thus each zooplankton sample represents a single “snapshot” of the system. Consequently, other important factors such as plankton succession [70, 87, 88], top-down control by fish [89, 90], and interactions among multiple variables, were not considered in our analyses. The fish community present in a waterbody can have a large impact on zooplankton dynamics [91–93], which likely played a role in the waterbody type differences in zooplankton densities found in this study. We also did not consider effects of elevation differences between waterbody types on crustacean zooplankton communities, which could impact our results [94]; however, the elevational difference between natural lakes and reservoirs was relatively similar in waterbodies in this study (natural lakes: 580 ± 650 m (1 S.D.); reservoirs: 740 ± 777 m). Lastly, we acknowledge that the vertical tow mesh size (0.243 mm) used in the waterbodies in the 2007 NLA may be large enough to miss smaller crustacean zooplankton taxa, especially in warmer regions of the U.S. Also, shorter WRT systems may contain smaller-bodied zooplankton that are removed from the system more quickly and are subsequently not able to reach as large of body sizes as in longer WRT systems [48, 66]. Therefore, we emphasize that our results are limited to crustacean zooplankton taxa retained by the 243-mm mesh. However, because calanoid taxa are generally much larger than many cladoceran taxa [1, 64], and juvenile and adult calanoids are larger (e.g., >0.5–1 mm in length) than the mesh size used in sampling, the overall result that calanoid density was greater in natural lakes than in reservoirs is supported. Furthermore, by including the larger-bodied cladoceran *Daphnia* in analyses, we were still able to compare calanoid copepods with one cladoceran taxa that was likely minimally affected by the sampling methods.

This study constitutes one of the most comprehensive spatial datasets for the analysis of zooplankton, spanning more than 700 waterbodies in the U.S. Altogether, our findings indicated differences in zooplankton community structure between natural lakes and reservoirs. Our results also indicated that the relative importance of environmental drivers on zooplankton communities varied between reservoirs and natural lakes. Such zooplankton community and density differences may in turn alter freshwater food webs and water quality [4, 5–7]. As the construction of reservoirs for many different human purposes increases in many regions of the world [83, 95], so does the need to better understand the resulting consequences of reservoir characteristics on plankton community dynamics, food webs, and water quality.

## Supporting information

S1 Table Pearson product-moment correlations of focal environmental variables included in the analyses. “NS” refers to relationships that are not significant and “.” refers to empty cells. The focal environmental variables included in our analyses were: Temp max = maximum waterbody temperature (°C), DO mean = mean water column dissolved oxygen concentration (mg L^-1^), Chlorophyll *a* = chlorophyll *a* concentration (μg L^-1^), and WRT = water residence time (years). Also included are other environmental variables not included in our analyses because of correlations with *r* > 0.50 with the focal environmental variables: Max depth = maximum waterbody depth (m), Calcium = calcium concentration (mg L^-1^), and DOC = dissolved organic carbon concentration (mg L^-1^).
(DOCX)

S2 Table Regression tree statistics for crustacean, total copepod, calanoid, and cladoceran density for all waterbodies, for just natural lakes, and for just reservoirs in the continental U.S. Reported for each regression analysis is each individual leaf group, *N* for each leaf, each leaf’s response variable mean ± SE, and the description for node splits for each leaf group. The percentage of the waterbodies that consist of reservoirs are provided for each leaf split, for analyses across both natural lakes and reservoirs.
(DOCX)

S3 Table The variable importance of the focal environmental variables provided for all regression analyses. All refers to aggregated analyses with both natural lakes and reservoirs. Temp max = maximum waterbody temperature (°C), DO mean = mean water column dissolved oxygen concentration (mg L^-1^), Chlorophyll *a* = chlorophyll *a* concentration (μg L^-1^), and WRT = water residence time (years).
(DOCX)

S1 Fig Location of reservoirs with different primary purposes across the U.S. Primary purpose was obtained for all reservoirs with available data from the U.S. Army Corps of Engineers’ National Inventory of Dams database. *N* > 20 for each of these categories.
(TIF)

S2 Fig Comparisons of a) crustacean zooplankton (copepod + cladoceran), b) total copepod (cyclopoid + calanoid), c) calanoid, and d) cladoceran, and e) crustacean zooplankton genera richness between natural lakes and reservoirs across the U.S. (TIF)

S3 Fig Locations of the regression analysis splits for calanoid zooplankton density across both natural lakes and reservoirs (*N* = 688). A refers to waterbodies that have pH ≤ 7.9; B refers to waterbodies with pH > 7.9 but ≤ 8.7; C refers to waterbodies with pH > 8.7 and water residence times > 0.338 years; D refers to waterbodies with pH > 8.7 and water residence times < 0.338 years.
(TIF)

S4 Fig Locations of the regression analysis splits for cladoceran zooplankton density across both natural lakes and reservoirs (*N* = 688). A refers to waterbodies that have maximum temperatures > 27.5 °C; B refers to waterbodies with maximum temperature < 27.5 °C and pH < 8.38; C refers to waterbodies with maximum temperature < 27.5 °C, pH > 8.38, and water residence times > 0.177 years; D refers to waterbodies with maximum temperature < 27.5 °C, pH > 8.38, and water residence times < 0.177 years.
(TIF)
