# Supplementary material for: Calanoid copepod zooplankton density is positively associated with water residence time across the continental United States
Source: PLoS One. 2019 Jan 9;14(1):e0209567. doi: 10.1371/journal.pone.0209567 (PMC6326432; doi:10.1371/journal.pone.0209567)
Supplement: S1 Table — “NS” refers to relationships that are not significant and “.” refers to empty cells. The focal environmental variables included in our analyses were: Temp max = maximum waterbody temperature (°C), DO mean = mean water column dissolved oxygen concentration (mg L-1), Chlorophyll a = chlorophyll a concentration (μg L-1), and WRT = water residence time (years). Also included are other environmental variables not included in our analyses because of correlations with r > 0.50 with the focal environmental variables: Max depth = maximum waterbody depth (m), Calcium = calcium concentration (mg L-1), and DOC = dissolved organic carbon concentration (mg L-1). (DOCX) [file pone.0209567.s001.docx]

| Variables | Temp max | DO mean | pH | Chlorophyll *a* | WRT | Max depth | Calcium | DOC |
| --- | --- | --- | --- | --- | --- | --- | --- | --- |
| Temp max | 1.00 | . | . | . | . | . | . | . |
| DO mean | -0.34 | 1.00 | . | . | . | . | . | . |
| pH | 0.12 | NS | 1.00 | . | . | . | . | . |
| Chlorophyll *a* | 0.38 | -0.13 | 0.38 | 1.00 | . | . | . | . |
| WRT | -0.22 | NS | 0.11 | -0.21 | 1.00 |  | . | . |
| Max depth | -0.17 | -0.16 | -0.17 | -0.52 | 0.43 | 1.00 | . | . |
| Calcium | 0.11 | NS | 0.74 | 0.34 | NS | -0.12 | 1.00 | . |
| DOC | 0.09 | NS | 0.45 | 0.52 | 0.11 | -0.55 | 0.34 | 1.00 |
