# Supplementary material for: Calanoid copepod zooplankton density is positively associated with water residence time across the continental United States
Source: PLoS One. 2019 Jan 9;14(1):e0209567. doi: 10.1371/journal.pone.0209567 (PMC6326432; doi:10.1371/journal.pone.0209567)
Supplement: S2 Table — Reported for each regression analysis is each individual leaf group, N for each leaf, each leaf’s response variable mean ± SE, and the description for node splits for each leaf group. The percentage of the waterbodies that consist of reservoirs are provided for each leaf split, for analyses across both natural lakes and reservoirs. (DOCX) [file pone.0209567.s002.docx]

| Response variable | Waterbody type | Leaf ID | *N* | Response variable mean ± SE | Leaf description | Percentage reservoirs |
| --- | --- | --- | --- | --- | --- | --- |
| Crustacean zooplankton density |  |  |  |  |  |  |
|  | All | A | 125 | 6.2 ± 1.6 | Maximum temperature greater than 27.55 ºC | 67% |
|  | All | B | 416 | 8.3 ± 1.0 | Maximum temperature less than 27.55 ºC; pH less than 8.655 and chlorophyll *a* less than 36.73 µg/L | 51% |
|  | All | C | 47 | 29.2 ± 7.0 | Maximum temperature less than 27.55 ºC; pH less than 8.655; chlorophyll *a* greater than 36.73 µg/L | 60% |
|  | All | D | 91 | 48.6 ± 6.7 | Maximum temperature less than 27.55 ºC; pH greater than 8.655; water residence time longer than 0.285 years | 21% |
|  | All | E | 9 | 6.0 ± 2.1 | Maximum temperature less than 27.55 ºC; pH greater than 8.655; water residence time shorter than 0.285 years | 60% |
|  |  |  |  |  |  |  |
|  | Natural lakes | A | 27 | 5.7 ± 4.2 | Maximum temperature greater than 28.02 ºC |  |
|  | Natural lakes | B | 178 | 6.7 ± 1.7 | Maximum temperature less than 28.02 ºC; pH less than 8.645; chlorophyll *a* less than 35.06 µg/L |  |
|  | Natural lakes | C | 16 | 29.4 ± 7.5 | Maximum temperature less than 28.02 ºC; pH less than 8.645; chlorophyll *a* greater than 35.06 µg/L |  |
|  | Natural lakes | D | 76 | 47.4 ± 6.7 | Maximum temperature less than 28.02 ºC; pH greater than 8.645 |  |
|  |  |  |  |  |  |  |
|  | Reservoirs | A | 126 | 6.6 ± 1.4 | Maximum temperature greater than 26.57 ºC |  |
|  | Reservoirs | B | 160 | 11.2 ± 1.8 | Maximum temperature less than 26.57 ºC; chlorophyll *a* less than 38.47 µg/L; water residence time longer than 0.330 years |  |
|  | Reservoirs | C | 72 | 8.1 ± 1.7 | Maximum temperature less than 26.57 ºC; chlorophyll *a* less than 38.47 µg/L; water residence time shorter than 0.330 years |  |
|  | Reservoirs | D | 33 | 43.9 ± 13.5 | Maximum temperature less than 26.57 ºC; chlorophyll *a* greater than 38.47 µg/L |  |
|  |  |  |  |  |  |  |
| Total copepod density |  |  |  |  |  |  |
|  | All | A | 111 | 2.0 ± 0.6 | pH less than 8.705; maximum temperature greater than 27.55 ºC |  |
|  | All | B | 425 | 3.3 ± 0.7 | pH less than 8.705; maximum temperature less than 27.55 ºC; chlorophyll *a* less than 36.73 µg/L |  |
|  | All | C | 50 | 17.5 ± 4.3 | pH less than 8.705; maximum temperature less than 27.55 ºC; chlorophyll *a* greater than 36.73 µg/L |  |
|  | All | D | 94 | 25.3 ± 4.9 | pH greater than 8.705; water residence time longer than 0.181 years |  |
|  | All | E | 8 | 4.4 ± 2.1 | pH greater than 8.705; water residence time shorter than 0.181 years |  |
|  |  |  |  |  |  |  |
|  | Natural lakes | A | 17 | 0.6 ± 0.2 | pH less than 8.645; chlorophyll *a* less than 105.5 µg/L; maximum temperature greater than 28.48 ºC |  |
|  | Natural lakes | B | 189 | 4.2 ± 1.6 | pH less than 8.645; chlorophyll *a* less than 105.5 µg/L; maximum temperature less than 28.48 ºC |  |
|  | Natural lakes | C | 9 | 22.7 ± 8.3 | pH less than 8.645; chlorophyll *a* greater than 105.5 µg/L |  |
|  | Natural lakes | D | 14 | 3.7 ± 1.4 | pH greater than 8.645; maximum temperature greater than 26.75 ºC |  |
|  | Natural lakes | E | 68 | 28.1 ± 4.6 | pH greater than 8.645; maximum temperature less than 26.75 ºC |  |
|  |  |  |  |  |  |  |
|  | Reservoirs | A | 116 | 2.7 ± 0.6 | Maximum temperature greater than 26.79 ºC |  |
|  | Reservoirs | B | 83 | 1.6 ± 0.3 | Maximum temperature less than 26.79 ºC; chlorophyll *a* less than 38.47 µg/L; pH less than 7.895 |  |
|  | Reservoirs | C | 156 | 4.1 ± 0.6 | Maximum temperature less than 26.79 ºC; chlorophyll *a* less than 38.47 µg/L; pH greater than 7.895 |  |
|  | Reservoirs | D | 36 | 23.6 ± 10.5 | Maximum temperature less than 26.79 ºC; chlorophyll *a* greater than 38.47 µg/L |  |
|  |  |  |  |  |  |  |
|  |  |  |  |  |  |  |
| Calanoid density | All | A | 241 | 1.0 ± 0.1 | pH less than 7.905 | 52% |
|  | All | B | 345 | 3.7 ± 0.8 | pH between 7.905 and 8.705 | 57% |
|  | All | C | 84 | 18.7 ± 3.3 | pH greater than 8.705; water residence time longer than 0.338 years | 19% |
|  | All | D | 18 | 2.6 ± 0.9 | pH greater than 8.705; water residence time shorter than 0.338 years | 54% |
|  |  |  |  |  |  |  |
|  | Natural lakes | A | 215 | 3.0 ± 1.0 | pH less than 8.645 |  |
|  | Natural lakes | B | 10 | 1.6 ± 0.8 | pH greater than 8.645; mean DO less than 4.3 mg/L |  |
|  | Natural lakes | C | 32 | 8.4 ± 2.4 | pH greater than 8.645; mean DO greater than 4.3 mg/L; maximum temperature greater than 24.1 ºC |  |
|  | Natural lakes | D | 40 | 31.5 ± 6.0 | pH greater than 8.645; mean DO greater than 4.3 mg/L; maximum temperature less than 24.1 ºC |  |
|  |  |  |  |  |  |  |
|  | Reservoirs | A | 22 | 0.4 ± 0.2 | pH less than 7.905; maximum temperature greater than 18.35 ºC |  |
|  | Reservoirs | B | 119 | 1.0 ± 0.2 | pH less than 7.905; maximum temperature less than 18.35 ºC |  |
|  | Reservoirs | C | 209 | 3.8 ± 0.8 | pH greater than 7.905; water residence time longer than 0.173 years |  |
|  | Reservoirs | D | 41 | 1.1 ± 0.4 | pH greater than 7.905; water residence time shorter than 0.173 years |  |
|  |  |  |  |  |  |  |
| Cladoceran density | All | A | 125 | 3.9 ± 1.1 | Maximum temperature greater than 27.55 °C |  |
|  | All | B | 311 | 5.4 ± 0.8 | Maximum temperature less than 27.55 °C; pH less than 8.38 |  |
|  | All | C | 237 | 12.6 ± 1.7 | Maximum temperature less than 27.55 °C; pH greater than 8.38; water residence time longer than 0.177 years |  |
|  | All | D | 15 | 1.4 ± 0.6 | Maximum temperature less than 27.55 °C; pH greater than 8.38; water residence time shorter than 0.177 years |  |
|  |  |  |  |  |  |  |
|  | Natural lakes | A | 24 | 4.8 ± 4.2 | Maximum temperature greater than 28.48 °C |  |
|  | Natural lakes | B | 156 | 2.8 ± 0.4 | Maximum temperature less than 28.48 °C; pH less than 8.485 |  |
|  | Natural lakes | C | 117 | 16.0 ± 3.0 | Maximum temperature less than 28.48 °C; pH greater than 8.485 |  |
|  |  |  |  |  |  |  |
|  | Reservoirs | A | 91 | 3.6 ± 1.1 | Maximum temperature greater than 27.55 °C |  |
|  | Reservoirs | B | 49 | 5.2 ± 3.4 | Maximum temperature less than 27.55 °C; mean DO less than 4.4 mg/L |  |
|  | Reservoirs | C | 251 | 8.5 ± 1.0 | Maximum temperature less than 27.55 °C; mean DO greater than 4.4 mg/L |  |
|  |  |  |  |  |  |  |
| *Daphnia* density | All | A | 125 | 0.8 ± 0.3 | Maximum temperature greater than 27.55 °C |  |
|  | All | B | 87 | 1.3 ± 0.3 | Maximum temperature less than 27.55 °C; pH less than 7.295 |  |
|  | All | C | 476 | 4.5 ± 0.5 | Maximum temperature less than 27.55 °C; pH greater than 7.295 |  |
|  |  |  |  |  |  |  |
|  | Natural lakes | A | 24 | 1.2 ± 1.2 | Maximum temperature greater than 28.48 °C |  |
|  | Natural lakes | B | 18 | 0.2 ± 0.1 | Maximum temperature less than 28.48 °C; pH less than 6.685 |  |
|  | Natural lakes | C | 255 | 3.9 ± 0.6 | Maximum temperature less than 28.48 °C; pH greater than 6.685 |  |
|  |  |  |  |  |  |  |
|  | Reservoir | A | 127 | 1.0 ± 0.1 | Maximum temperature greater than 26.52 °C |  |
|  | Reservoir | B | 40 | 1.2 ± 0.4 | Maximum temperature less than 26.52 °C; pH less than 7.435 |  |
|  | Reservoir | C | 224 | 5.2 ± 1.0 | Maximum temperature less than 26.52 °C; pH greater than 7.435 |  |
