# Supplementary material for: Calanoid copepod zooplankton density is positively associated with water residence time across the continental United States
Source: PLoS One. 2019 Jan 9;14(1):e0209567. doi: 10.1371/journal.pone.0209567 (PMC6326432; doi:10.1371/journal.pone.0209567)
Supplement: S3 Table — All refers to aggregated analyses with both natural lakes and reservoirs. Temp max = maximum waterbody temperature (°C), DO mean = mean water column dissolved oxygen concentration (mg L-1), Chlorophyll a = chlorophyll a concentration (μg L-1), and WRT = water residence time (years). (DOCX) [file pone.0209567.s003.docx]

| Response variable | Waterbody type | Explanatory variable | Variable importance |
| --- | --- | --- | --- |
| Crustacean zooplankton  density |  |  |  |
|  | All | Temp max | 46 |
|  | All | pH | 29 |
|  | All | Chlorophyll *a* | 14 |
|  | All | WRT | 9 |
|  | All | DO mean | 2 |
|  |  |  |  |
|  | Natural lakes | Temp max | 41 |
|  | Natural lakes | pH | 34 |
|  | Natural lakes | Chlorophyll *a* | 22 |
|  | Natural lakes | DO mean | 3 |
|  | Natural lakes | WRT | 0 |
|  |  |  |  |
|  | Reservoirs | Temp max | 50 |
|  | Reservoirs | Chlorophyll *a* | 19 |
|  | Reservoirs | WRT | 19 |
|  | Reservoirs | DO mean | 12 |
|  | Reservoirs | pH | 1 |
|  |  |  |  |
| Total copepod density |  |  |  |
|  | All | pH | 41 |
|  | All | Chlorophyll *a* | 23 |
|  | All | Temp max | 22 |
|  | All | WRT | 13 |
|  | All | DO mean | 1 |
|  |  |  |  |
|  | Natural lakes | pH | 43 |
|  | Natural lakes | Chlorophyll *a* | 27 |
|  | Natural lakes | Temp max | 24 |
|  | Natural lakes | DO mean | 4 |
|  | Natural lakes | WRT | 3 |
|  |  |  |  |
|  | Reservoirs | Temp max | 43 |
|  | Reservoirs | Chlorophyll *a* | 26 |
|  | Reservoirs | pH | 19 |
|  | Reservoirs | DO mean | 9 |
|  | Reservoirs | WRT | 3 |
|  |  |  |  |
| Calanoid density | All | pH | 71 |
|  | All | WRT | 16 |
|  | All | Chlorophyll *a* | 8 |
|  | All | Temp max | 4 |
|  | All | DO mean | 1 |
|  |  |  |  |
|  | Natural lakes | pH | 48 |
|  | Natural lakes | DO mean | 21 |
|  | Natural lakes | Chlorophyll *a* | 18 |
|  | Natural lakes | Temp max | 10 |
|  | Natural lakes | WRT | 3 |
|  |  |  |  |
|  | Reservoirs | pH | 40 |
|  | Reservoirs | WRT | 29 |
|  | Reservoirs | Temp max | 28 |
|  | Reservoirs | Chlorophyll *a* | 3 |
|  | Reservoirs | DO mean | 0 |
|  |  |  |  |
|  |  |  |  |
| Cladoceran density | All | Temp max | 73 |
|  | All | WRT | 12 |
|  | All | pH | 11 |
|  | All | Chlorophyll *a* | 3 |
|  | All | DO mean | 2 |
|  |  |  |  |
|  | Natural lakes | Temp max | 82 |
|  | Natural lakes | pH | 12 |
|  | Natural lakes | Chlorophyll *a* | 5 |
|  | Natural lakes | DO mean | 0 |
|  | Natural lakes | WRT | 0 |
|  |  |  |  |
|  | Reservoirs | Temp max | 71 |
|  | Reservoirs | DO mean | 27 |
|  | Reservoirs | Chlorophyll *a* | 1 |
|  | Reservoirs | WRT | 1 |
|  | Reservoirs | pH | 0 |
|  |  |  |  |
|  |  |  |  |
| *Daphnia* density | All | Temp max | 75 |
|  | All | pH | 23 |
|  | All | DO mean | 1 |
|  | All | Chlorophyll *a* | 1 |
|  | All | WRT | 0 |
|  |  |  |  |
|  | Natural lakes | Temp max | 74 |
|  | Natural lakes | pH | 26 |
|  | Natural lakes | DO mean | 0 |
|  | Natural lakes | Chlorophyll *a* | 0 |
|  | Natural lakes | WRT | 0 |
|  |  |  |  |
|  | Reservoirs | Temp max | 66 |
|  | Reservoirs | DO mean | 16 |
|  | Reservoirs | pH | 14 |
|  | Reservoirs | WRT | 3 |
|  | Reservoirs | Chlorophyll *a* | 1 |
