# Supplementary figures and images for: Calanoid copepod zooplankton density is positively associated with water residence time across the continental United States
Source: PLoS One. 2019 Jan 9;14(1):e0209567. doi: 10.1371/journal.pone.0209567 (PMC6326432; doi:10.1371/journal.pone.0209567)

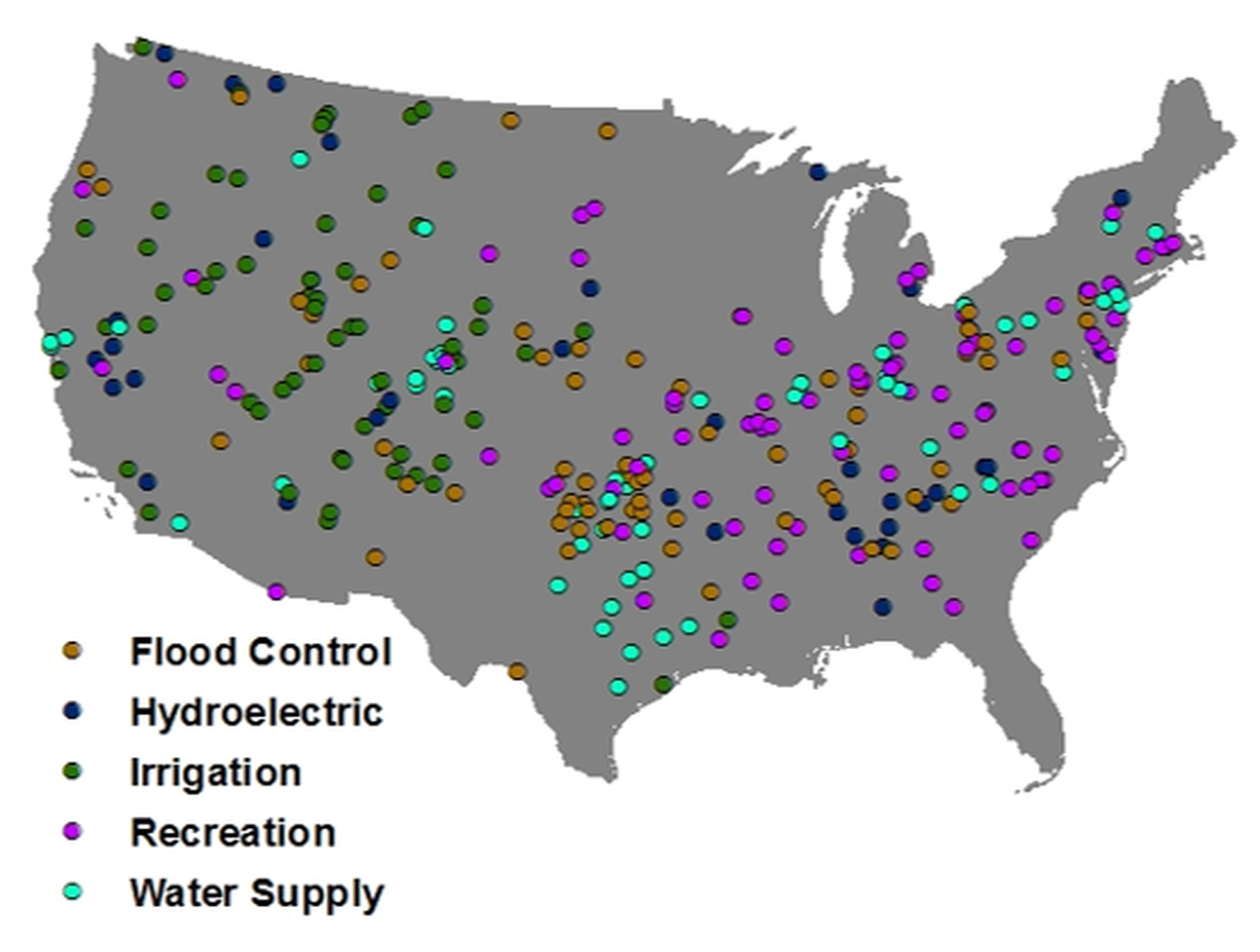

Supplement: S1 Fig — Primary purpose was obtained for all reservoirs with available data from the U.S. Army Corps of Engineers’ National Inventory of Dams database. N > 20 for each of these categories. (TIF) [file pone.0209567.s004.tif]

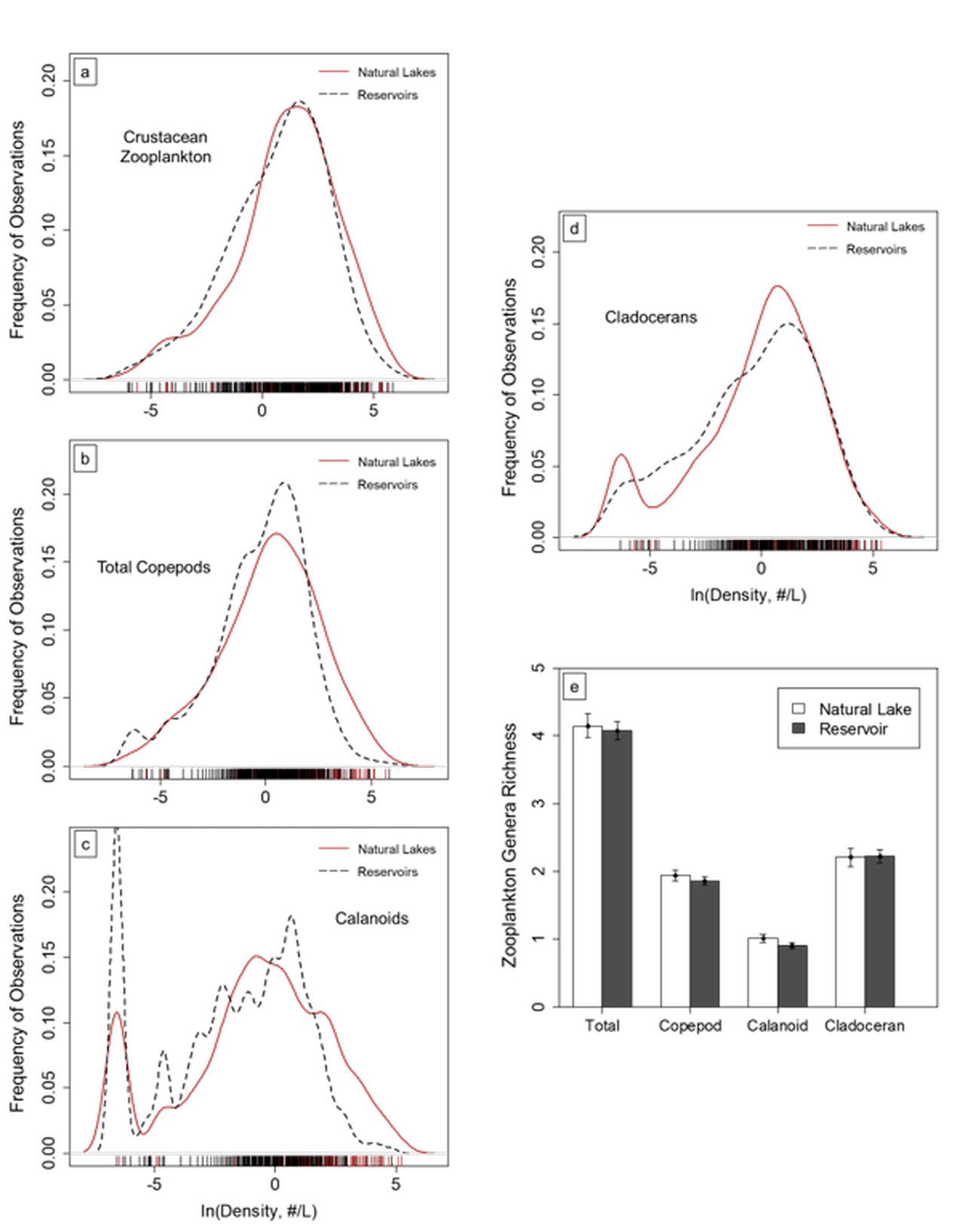

Supplement: S2 Fig — (TIF) [file pone.0209567.s005.tif]

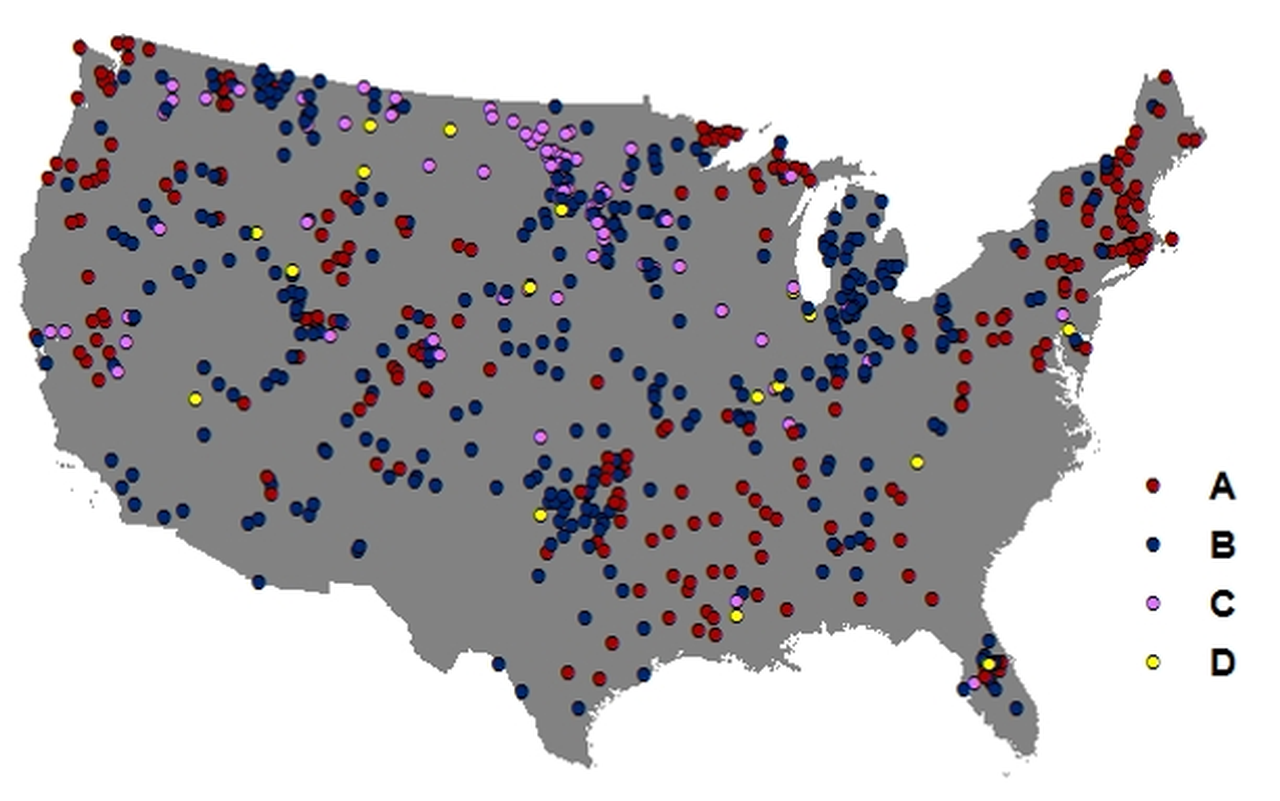

Supplement: S3 Fig — A refers to waterbodies that have pH ≤ 7.9; B refers to waterbodies with pH > 7.9 but ≤ 8.7; C refers to waterbodies with pH > 8.7 and water residence times > 0.338 years; D refers to waterbodies with pH > 8.7 and water residence times < 0.338 years. (TIF) [file pone.0209567.s006.tif]

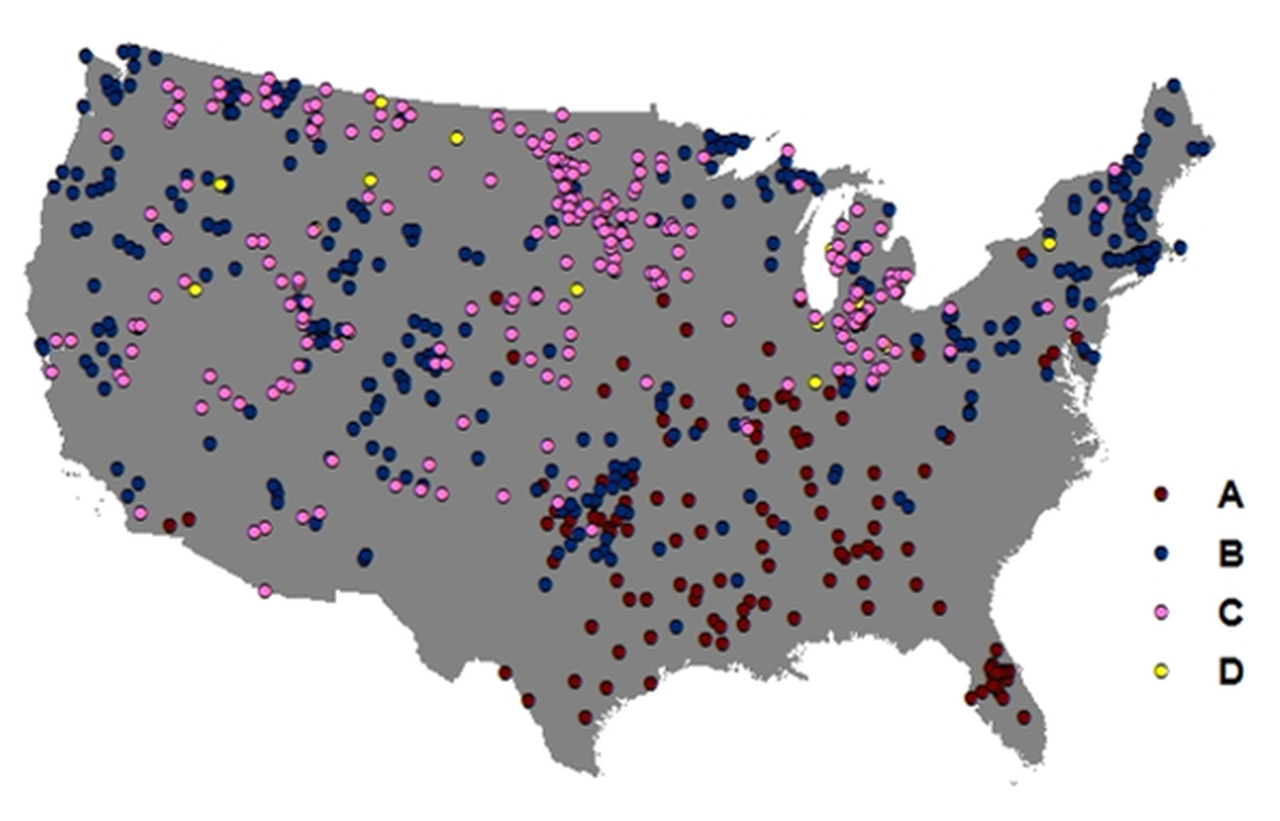

Supplement: S4 Fig — A refers to waterbodies that have maximum temperatures > 27.5 °C; B refers to waterbodies with maximum temperature < 27.5 °C and pH < 8.38; C refers to waterbodies with maximum temperature < 27.5 °C, pH > 8.38, and water residence times > 0.177 years; D refers to waterbodies with maximum temperature < 27.5 °C, pH > 8.38, and water residence times < 0.177 years. (TIF) [file pone.0209567.s007.tif]
